# Design and demonstration of an underwater acoustic carpet cloak

**DOI:** 10.1038/s41598-017-00779-4

**Published:** 2017-04-06

**Authors:** Yafeng Bi, Han Jia, Wenjia Lu, Peifeng Ji, Jun Yang

**Affiliations:** 1 Key Laboratory of Noise and Vibration Research, Institute of Acoustics, Chinese Academy of Sciences, Beijing, 100190 People’s Republic of China; 2 University of Chinese Academy of Sciences, Beijing, 100049 People’s Republic of China; 3 State Key Laboratory of Acoustics, Institute of Acoustics, Chinese Academy of Sciences, Beijing, 100190 People’s Republic of China

## Abstract

The carpet cloak, which is designed to hide the objects placed on a reflecting surface, has become a topic of considerable interest. Inspired by those theoretical works, the experimental realization of acoustic carpet cloak in air host has been reported. However, due to the difficulty in obtaining the unit cell in reality, the underwater carpet cloak still remains in simulation thus far. Here, we design and fabricate a realizable underwater acoustic carpet cloak. By introducing a scaling factor, the structure of the carpet cloak, which is comprised of layered brass plates, is greatly simplified at the cost of some impedance match. The experimental results demonstrate a good performance of the proposed carpet cloak in a wide frequency range. Our work paves the way for future applications in the practical underwater devices.

## Introduction

Transformation acoustics, which is introduced to design new acoustic structures, shows the way to control the propagation of acoustic waves^1–5^. Invisibility cloak is one of the most significant applications in transformation acoustics. It has attracted much attention in the past few years^6, 7^. The acoustic cloak is a material shell that can control the sound wave propagating direction to make the target undetectable in acoustic system. The parameters of the cloak shell can be given by transformation acoustics. Unfortunately, in most cases, these parameters are very complex: the space-dependent mass density and bulk modulus are usually inhomogeneous and extremely anisotropic. As a result, these parameters are challenging to achieve in practice.

Subsequent research leads to the concept of carpet cloak^8–40^, which is proven to be practically feasible. The carpet cloak is able to hide the target placed on a reflecting surface. The device modifies the acoustic signature of the target and mimics the acoustic field obtained from a reflecting plane, so that the cloaked target is indistinguishable from the reflecting surface. The concept of the carpet cloak was firstly introduced in context of electromagnetics^8–20^ and rapidly extended to acoustics^21–31^. It was firstly proposed as a quasiconformal carpet cloak^8–15^. However, the size of the quasiconformal carpet cloak is quite large: usually it is an order of magnitude larger than the target. Besides, the lateral shift, which comes from the neglect of the weak anisotropy, will make it possible to be detectable^32^.

To get rid of these disadvantages, the carpet cloak with linear transformation was proposed^20, 21^. The linear transformation from a bump in the physical space to a plane in the virtual space brings homogeneous parameters with reasonable anisotropy, which are much more practical for realization. By using the layered perforated plates, both the two-dimensional (2D) and 3D acoustic carpet cloaks were realized in the air host^22–27^. However, because of the difficulty in obtaining the assumed materials, the underwater carpet cloak still remains in simulation^28, 29^.

In this article, we present a design approach of an underwater carpet cloak which is much easier to realize. By introducing a scaling factor, a modest impedance mismatch is brought in to simplify the structure of the carpet cloak. The quasi-two-dimensional device, which is made up of layered brass plates, has surprising low complexity. We verify our method by designing and fabricating a realizable acoustic carpet cloak, and investigate its effectiveness through experiments. Its good performance demonstrates that the cloak can work stably in a wide frequency range.

## Results

### Coordinate transformation and prototype for underwater carpet cloak

**Figure 1:**
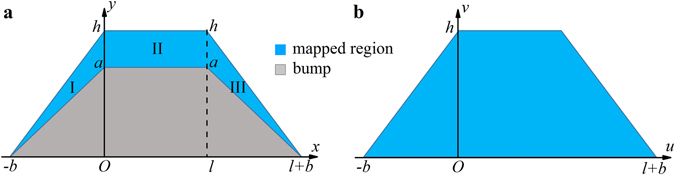
Schematic of the transformation for the carpet cloak. (**a**) The physical space whose coordinate frame is (*x*, *y*, *z*). The cloaked bump is defined by the grey trapezoid. The carpet cloak (blue region) is separated into two triangles (region I and region III) and one rectangle (region II). (**b**) The virtual space whose coordinate frame is (*u*, *v*, *w*). The blue region in the physical space (**a**) is mapped to the blue trapezoid in the virtual space (**b**) by the linear transformation.

A schematic of the 2D carpet cloak model is shown in Fig. 1. In the physical space (Fig. 1a), a gray trapezoidal bump is placed on the ground plane. In order to make it invisible from the sound detection, the trapezoidal bump is covered with a blue cloak whose acoustic parameters are specified by the transformation acoustics theory. If we map the blue cloak region in the physical space to the whole trapezoidal region above the ground in the virtual space (Fig. 1b), the space mapping rules could be defined mathematically as follow:

1 $$\{\begin{array}{ll}u=x,v=\frac{h}{h-a}(-a+\frac{a}{b}|x|+y),w={\omega }_{1}z, & {\rm{region}}\,{\rm{I}}\\ u=x,v=\frac{h}{h-a}(y-a),w={\omega }_{2}z, & {\rm{region}}\,{\rm{II}}\\ u=x-l,v=\frac{h}{h-a}(-a+\frac{a}{b}(x-l)+y),w={\omega }_{3}z, & {\rm{region}}\,{\rm{III}}\end{array},$$

where *a*, *b*, *l*, *d* and *h* are geometric parameters of the model indicated in Fig. 1, and *ω* is an additional degree of freedom to scale the impedance mismatch between the transformed material and the background fluid^24, 26^. By utilizing these mapping rules, the whole trapezoid in the virtual space is compressed into two triangles (region I and region III) and one rectangle (region II) in the physical space. From Eq. (1), one can note that the mapping rules between the physical space and the virtual space are linear. These linear transformations will lead to homogeneous material parameters which are much more achievable. According to transformation acoustics^1, 2^, the mass density and bulk modulus distributions of the cloak can be given by:

2 $$\{\begin{array}{rcl}{\boldsymbol{\rho }} & = & \det (A){({A}^{-1})}^{T}({A}^{-1}){\rho }_{0}\\ K & = & {\rm{\det }}(A){K}_{0}\end{array},$$

where *ρ*_0_ and *K*_0_ are the density and the bulk modulus of background fluid respectively; *A* is the transformation Jacobian matrix showed as: ${A}=\frac{\partial (x,y,z)}{\partial (u,v,w)}$.

By using Eqs (1) and (2), the material parameters of the cloak can be obtained. For convenience, the off-diagonal terms in the mass density tensor are eliminated by rotating the coordinate axes anticlockwise with an angle *α*. Then the eigenvalues of the mass density tensor are presented as:

3 $$\{\begin{array}{ll}{K}^{I}=\frac{h-a}{h{\omega }_{1}}{K}_{0},{\rho }_{11}^{pr,I}=\frac{F-\sqrt{{F}^{2}-1}}{{\omega }_{1}}{\rho }_{0},{\rho }_{22}^{pr,I}=\frac{F+\sqrt{{F}^{2}-1}}{{\omega }_{1}}{\rho }_{0}, & {\rm{region}}\,{\rm{I}}\\ {K}^{II}=\frac{h-a}{h{\omega }_{2}}{K}_{0},{\rho }_{11}^{pr,II}=\frac{h-a}{h{\omega }_{2}}{\rho }_{0},{\rho }_{22}^{pr,II}=\frac{h}{(h-a){\omega }_{2}}{\rho }_{0}, & {\rm{region}}\,{\rm{II}}\\ {K}^{III}=\frac{h-a}{h{\omega }_{3}}{K}_{0},{\rho }_{11}^{pr,III}=\frac{F-\sqrt{{F}^{2}-1}}{{\omega }_{3}}{\rho }_{0},{\rho }_{22}^{pr,III}=\frac{F+\sqrt{{F}^{2}-1}}{{\omega }_{3}}{\rho }_{0}, & {\rm{region}}\,{\rm{III}}\end{array},$$

where ${F}=1+\frac{{a}^{2}({b}^{2}+{h}^{2})}{2{b}^{2}h(h-a)}$. All the eigenvalues of the mass density tensor are marked with the superscript *pr*. Meanwhile, the rotating angle *α* is given by:

4 $$\alpha =\{\begin{array}{ll}\frac{\pi }{2}-\arcsin (\frac{G}{\sqrt{{G}^{2}+1}}), & {\rm{region}}\,{\rm{I}}\\ 0, & {\rm{region}}\,{\rm{II}}\\ -\frac{\pi }{2}+\arcsin (\frac{G}{\sqrt{{G}^{2}+1}}), & {\rm{region}}\,{\rm{III}}\end{array},$$

where ${G}=\frac{h}{b}(1-\frac{h-a}{a}(F-1-\sqrt{{F}^{2}-1}))$. Actually, *α* is also the angle between the principal axes of the transformed materials and the coordinate axes in the physical space.

Then, a prototype is supposed to illustrate the design of the carpet cloak. The geometry parameters in Fig. 1 are set as *a* = 28.5 mm, *b* = 100 mm, *h* = 85 mm, *l* = 40 mm, respectively. Besides, *ω*_1_ = *ω*_2_ = *ω*_3_ = 1 indicates that the carpet cloak is impedance matched with the background in this model. By substituting these parameters into Eqs (3) and (4), the required parameters of the cloak can be obtained:

5 $$\{\begin{array}{ll}{\rho }_{11}^{{pr},{I}}=0.59{\rho }_{0},{\rho }_{22}^{pr,I}=1.70{\rho }_{0},{K}^{pr,I}=0.66{K}_{0},\alpha =25^\circ ,\,\, & {\rm{region}}\,{\rm{I}}\\ {\rho }_{11}^{{pr},{II}}=0.66{\rho }_{0},{\rho }_{22}^{pr,II}=1.50{\rho }_{0},{K}^{pr,II}=0.66{K}_{0},\alpha =0^\circ ,\, & {\rm{region}}\,{\rm{II}}\\ {\rho }_{11}^{pr,III}=0.59{\rho }_{0},{\rho }_{22}^{pr,III}=1.70{\rho }_{0},{K}^{pr,III}=0.66{K}_{0},\alpha =-25^\circ ,\,\, & {\rm{region}}\,{\rm{III}}\end{array},$$

**Figure 2:**
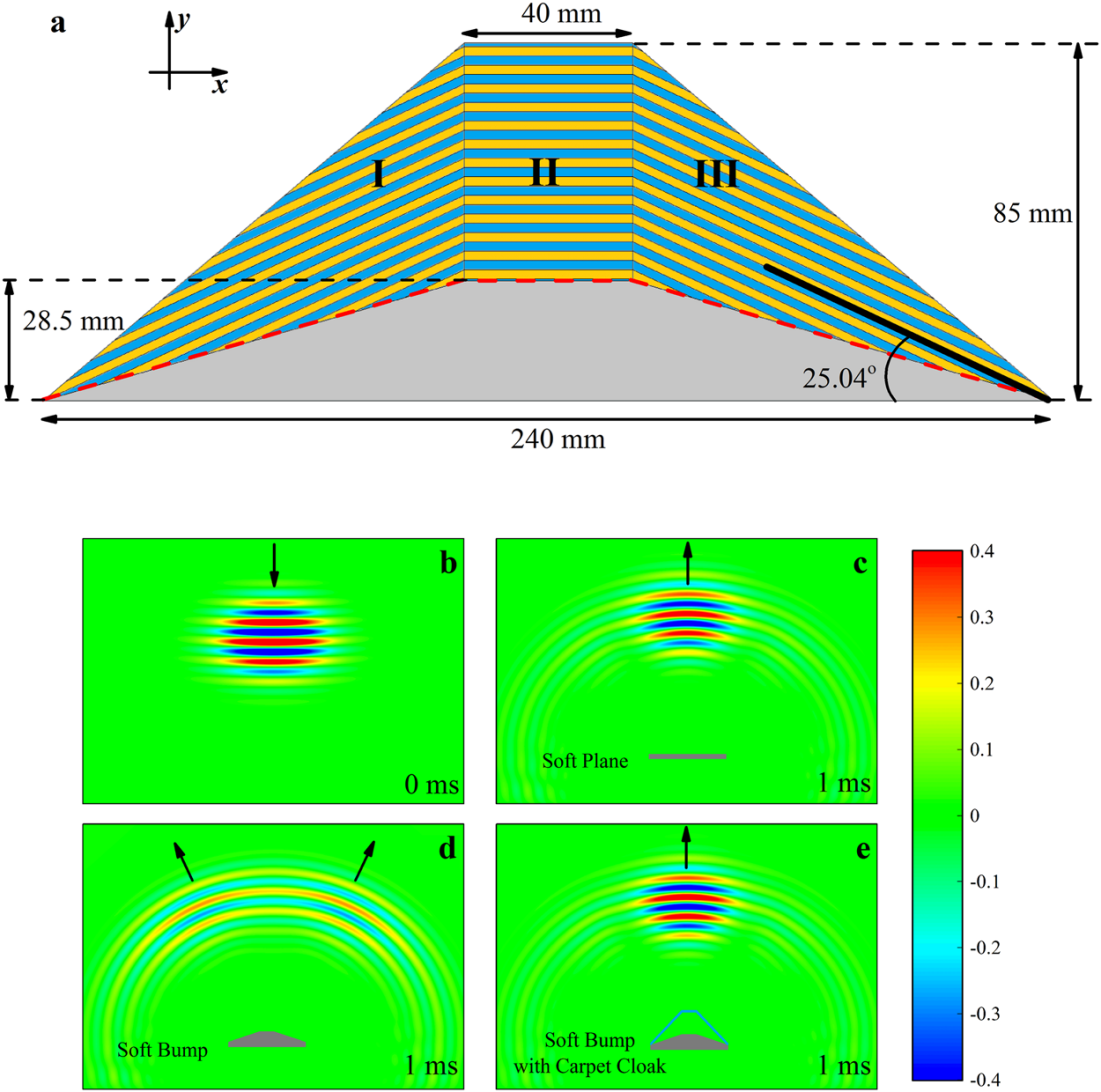
Numerical simulations of the underwater carpet cloak. (**a**) Schematic view of the ideal carpet cloak. Each region of the carpet cloak is a multilayered structure which is made up of two different materials (blue and yellow layers) with the same thickness. (**b**) The incident acoustic field when a Gaussian pulse signal is emitted from the top boundary. The background fluid is water. (**c**–**e**) The scattered acoustic fields. The acoustic wave is reflected by (**c**) a soft plane, (**d**) a soft bump and (**e**) a soft bump with the carpet cloak, respectively. Black arrows represent the propagation of the wave.

Obviously, material with anisotropic mass density is required in realizing the carpet cloak. It is known that there is no natural material with anisotropic mass density. Nevertheless, it has been demonstrated from the Biot fluid theory that layers of isotropic materials can present effective anisotropic mass density in long wavelength regime^4, 41^. If the thicknesses of the layers are much smaller than the wavelength, the acoustic layered system will have the following effective parameters:

6 $$\frac{1}{{\rho }_{11}}=\langle \frac{1}{{\rho }_{i}}\rangle ,{\rho }_{22}=\langle {\rho }_{i}\rangle ,\frac{1}{K}=\langle \frac{1}{{K}_{i}}\rangle ,$$

where 〈 〉 denotes a thickness weighted average; *ρ*_11_ (*ρ*_22_) is the effective mass density component in the direction which is parallel (perpendicular) to the layered structure (correspond to the eigenvalues of the mass density tensor in two principal axes); *ρ*_*i*_ and *K*_*i*_ are the dens_*i*_ty and bulk modulus in *i*-th layer. Therefore, the required material parameters in Eq. (5) can be obtained through the periodical layers. As shown in Fig. 2a, the designed carpet cloak is a multilayer structure, and it is comprised of two kinds of fluid (marked with A and B) with the same thickness. The blue regions represent layer A, the yellow regions represent layer B, and the gray region represents the area remaining to be concealed. The thickness of each layer is 1 mm, which is smaller than the wavelength at the operation frequency of 13 kHz by a factor of 40. The Biot fluid theory implies that there is an unique solution for the densities but infinite choices for the bulk moduli in layer A and layer B. For convenient comparison with the practical parameters of the designed sample discussed later, here we choose a special solution with the following parameters:

7 $$\{\begin{array}{rcl}{\rho }_{A}^{I}={\rho }_{A}^{III} & = & 0.33{\rho }_{0},{K}_{A}^{I}={K}_{A}^{III}=0.34{K}_{0}\\ {\rho }_{B}^{I}={\rho }_{B}^{III} & = & 3.07{\rho }_{0},{K}_{B}^{I}={K}_{B}^{III}=21.28{K}_{0}\\ {\rho }_{A}^{II} & = & 0.38{\rho }_{0},{K}_{A}^{II}=0.34{K}_{0}\\ {\rho }_{B}^{II} & = & 2.62{\rho }_{0},{K}_{B}^{II}=20.55{K}_{0}\end{array},$$

where the superscript I, II, III correspond to region I, II, III, respectively; the subscript A, B represent layer A and layer B.

### Numerical simulations

By utilizing the parameters in Eq. (7), the ideal cloak, whose impedance is matched with the background fluid, is designed and the camouflage effect of the proposed model is simulated in time domain with a commercial finite elements package (COMSOL Multiphysics). In simulation, the background fluid is water and the boundaries of the simulated area are set as absorbing boundaries to avoid unexpected reflections. Because the carpet cloak works with the reflecting plane, the material of all the scatterers is set as air to ensure strong impedance mismatch.

The simulated results are presented in Fig. 2b–e. In Fig. 2b, a short Gaussian pulse with central frequency of 13 kHz is emitted from the top boundary. The beam whose width is about 0.5 m directly propagates towards the targets. The time of Fig. 2b is set as 0 ms as a reference. Figure 2c–e show the acoustic fields in different cases after 1 ms. The short pulses arrive at the objects and then are reflected back. All the propagating directions of the wave are indicated by the black arrows. When the acoustic wave is reflected from a soft plane (Fig. 2c), the beam keeps its Gaussian shape and propagates from bottom to top in the backscattering direction. In contrast, Fig. 2d displays the acoustic pressure field obtained with the soft bump. It is obvious that there is a shadow in the middle of the scattered wavefront. The soft bump separates the beam into two parts. The widely scattered wave leads to a decrease of the energy density, which makes the amplitude of the scattered wave much smaller. Besides, the soft bump also causes a phase advance compared with the soft plane. However, by covering the soft bump with the carpet cloak, the scattered wave returns to the backscattering direction, as shown in Fig. 2e. The wave is modulated by the cloak. Its shape, amplitude and propagating direction are identical to those when the target is a soft plane. The cloaked object successfully mimics the reflecting plane and it is invisible under the sound detection.

### Design of the sample and experimental measurements

The simulated results for the prototype show the possibility to realize the carpet cloak. An ideal carpet cloak requires two kinds of materials: one (layer A) is much less dense but has the same acoustic velocity as water; the other (layer B) has much larger density and modulus. In fact, the much less dense fluid (layer A) is very challenging to achieve. Nevertheless, if some impedance mismatch (*ω* ≠ 1) is introduced into the carpet cloak, the materials become much more achievable. This change brings some undesired reflection but has limited impact on the camouflage effect.

For region I, we set *ω*_1_ = 0.34, which means that the mass density and bulk modulus of the cloak are 3 times those of water, then the scaled parameters of the cloak are ${\rho }_{11}^{pr,new}=3{\rho }_{11}^{pr}$, ${\rho }_{22}^{pr,new}=3{\rho }_{22}^{pr}$, ${K}^{pr,new}=3{K}^{pr}$. The material parameters in the layered structure are also changed:

8 $$\{\begin{array}{rcl}{\rho }_{A}^{I,new} & = & 0.97{\rho }_{0},{K}_{A}^{I,new}={K}_{0}\\ {\rho }_{B}^{I,new} & = & 9.03{\rho }_{0},{K}_{B}^{I,new}=62.59{K}_{0}\end{array}.$$

Now, above parameters can be realized by using water (layer A) and brass (layer B): the density and the acoustic velocity of water are 1000 kg/m^3^ and 1480 m/s; the Young’s modulus, density and Poisson’s ratio of the brass are 110 Gpa, 8900 kg/m^3^, and 0.35, respectively. It should be noted that these thin brass plates are separated by the water layers, so the influence of shear wave on the effective mass density can be neglected^28^. The thin brass plates could be approximately regarded as fluid at low frequency range.

**Figure 3:**
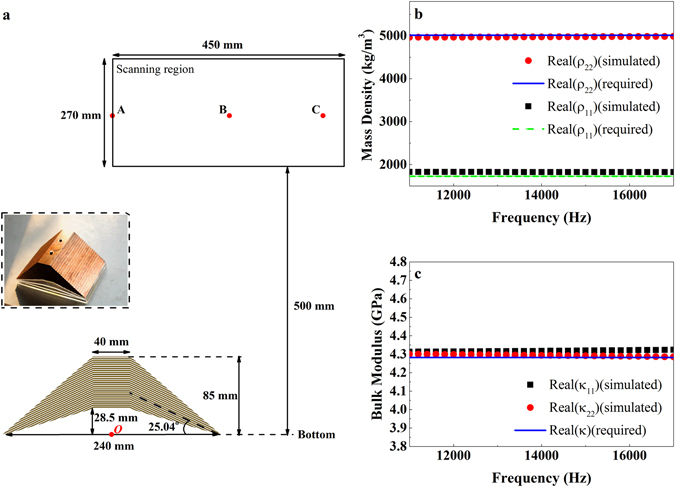
Experimental setup and prototype of the carpet cloak. (**a**) Schematics of the experimental setup and the sample. The scanned area is shown as the black frame. A, B and C are three particular points to extract the time domain signals. Inset: the photograph of the quasi-two-dimensional carpet cloak which consists of layered brass plates. Retrieved effective (**b**) mass density and (**c**) bulk modulus of the designed unit cell from 11kHz to 17 kHz in two principal axes. The horizontal lines in (**b** and **c**) show the parameters required by the cloak.

The effective acoustic parameters of the unit cell, which is comprised of brass and water, are calculated by using the retrieving method in simulation^42^. The results are presented in Fig. 3b and c. In Fig. 3b, the simulated mass density in two principal axes are plotted as symbols, while the required values obtained from the Biot theory are plotted as lines for comparison. The obvious different values in two directions indicate the anisotropic mass density. Similarly, the simulated (symbols) and required (line) bulk moduli in two principal axes are presented in Fig. 3c. The simulated values in two directions are almost the same. So the effective bulk modulus is isotropic in this unit cell. It can be seen that all these simulated results fit well with these required values in Fig. 3, which means the unit cell can work stably in a wide frequency range.

Due to the symmetry of the trapezoid, the parameters in region I and region III are the same. Actually, the parameters in region II can also be realized by the same structure for just a little change of the scaling factor (*ω*_2_ = 0.32). So the carpet cloak sample could be manufactured by the same unit cell (brass and water with filling rate *f*_brass_ = 0.5).

A photograph of the fabricated carpet cloak sample is shown in the inset of Fig. 3a. It’s a quasi-two-dimensional carpet cloak whose geometry size is the same with the simulated one, but with a thickness of 160 mm in z-direction. It comprises layers of brass plate with small channels filled with water.

The sample is placed in the middle of an anechoic water tank (about 2500 mm under water). An omnidirectional cylindrical transducer is placed above the sample (about 1300 mm away from the bottom of the sample, as shown in Fig. 3a). Two hydrophones (Type 8103, B&K) are used to measure the acoustic pressure fields. One is fixed in a position near the source as a time reference, and the other scans the measuring region step by step. The scanning hydrophone moves on a square grid of 15 mm to ensure at least six measurement points per wavelength. The scanned area, which is indicated as a black frame in Fig. 3a, is 450 mm wide and 270 mm high, and 500 mm away from the bottom of the cloak. The region is selected so that the incident wave and scattered wave can be separated clearly. All the emitting and receiving acoustic signals are analyzed by a multianalyzer system (Type 3160, B&K).

In order to distinguish the incident wave from the scattered wave, the omnidirectional cylindrical transducer emits short Gaussian pulses modulated with different sinusoidal signals (11 kHz to 16 kHz with 1 kHz step). To demonstrate the effectiveness of the cloak, three different cases were measured: with the soft plane (air layer sealed by polymethyl methacrylate) underwater, with the soft bump (air trapezoid sealed by polymethyl methacrylate) underwater and with the cloaked soft bump underwater, respectively.

**Figure 4:**
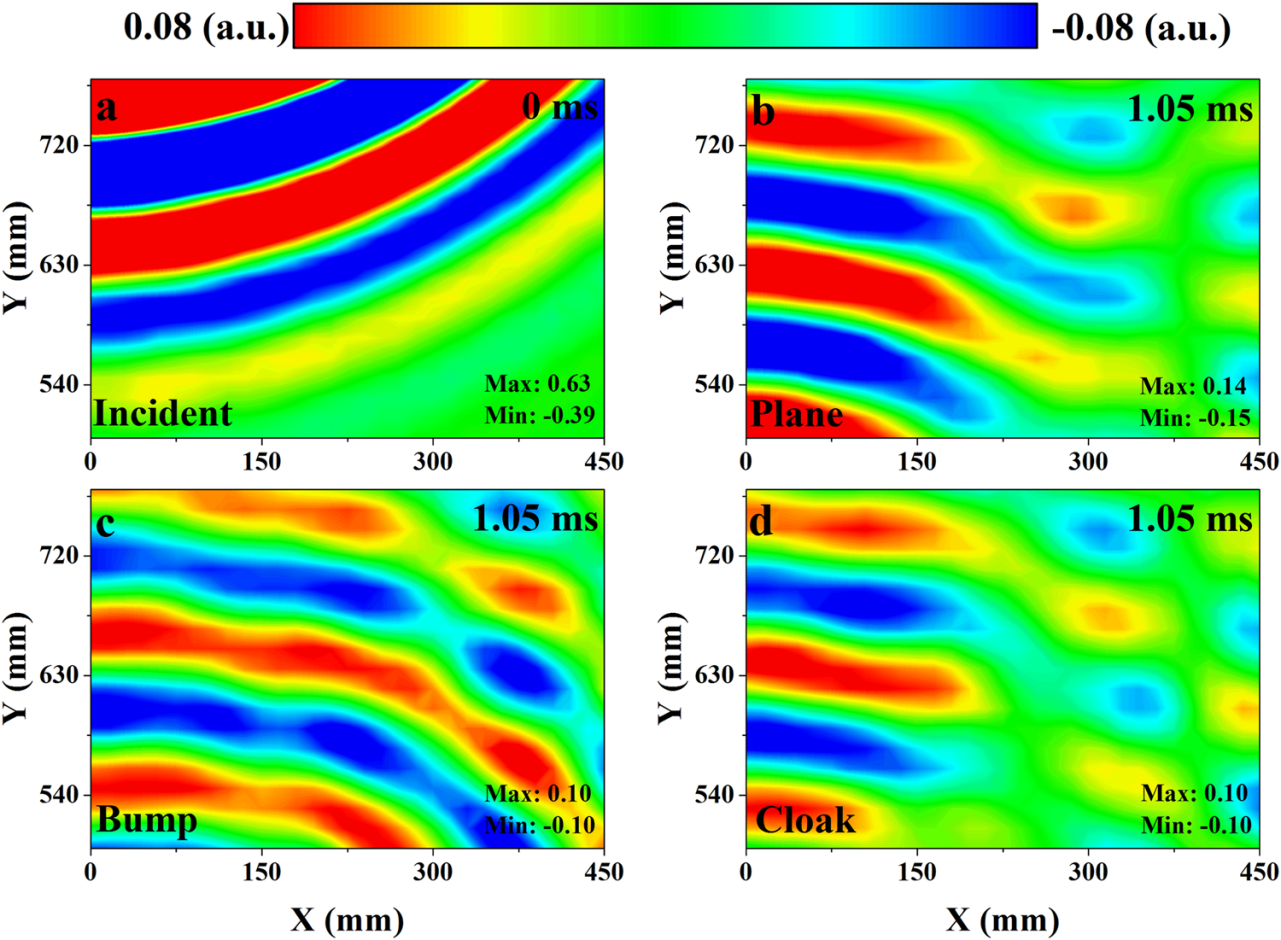
Measured acoustic pressure fields at 13 kHz. (**a**) The acoustic field of the incident pulse. The time of the incident acoustic field is set as 0 ms. (**b**–**d**) The scattered pressure fields measured after 1.05 ms. The short Gaussian pulse is reflected from the (**b**) soft plane, (**c**) soft bump and (**d**) soft bump covered by carpet cloak respectively.

The measured incident and scattered acoustic pressure fields at 13 kHz are presented in Fig. 4. The color scales of these fields have been tuned to make the distribution of energy and phase clear. In Fig. 4a, the source produces a pulse in the water and the wave spreads around from the upper left corner. After 1.05 ms, the wave is reflected by the object and propagates from bottom to the top. It can be observed from Fig. 4b that the reflected wave from the soft plane mainly focuses on the backscattering direction. In Fig. 4c, due to the slopes of the soft bump, acoustic wave obliquely hits the surfaces and reflects to the opposite sides. Consequently, the scattered wave inclines to both sides of the trapezoid. The scattered wave from the soft bump spreads evenly. This is very different from the propagating direction of scattered wave from the soft plane. In contrast, after covering the soft bump with cloak, the propagating direction of the scattered wave focuses on the backscattering direction again (Fig. 4d). The reflected wave from the cloaked soft bump is nearly identical to the wave from the soft plane as if the soft bump didn’t exist. The phase, the amplitude and the propagation direction of the scattered wave are recovered at the same time. These phenomena agree well with the simulations (see Supplementary Fig. S1). The simulated results also confirm the validity of the designed cloak at oblique incidence (see Supplementary Figs S2 and S3).

**Figure 5:**
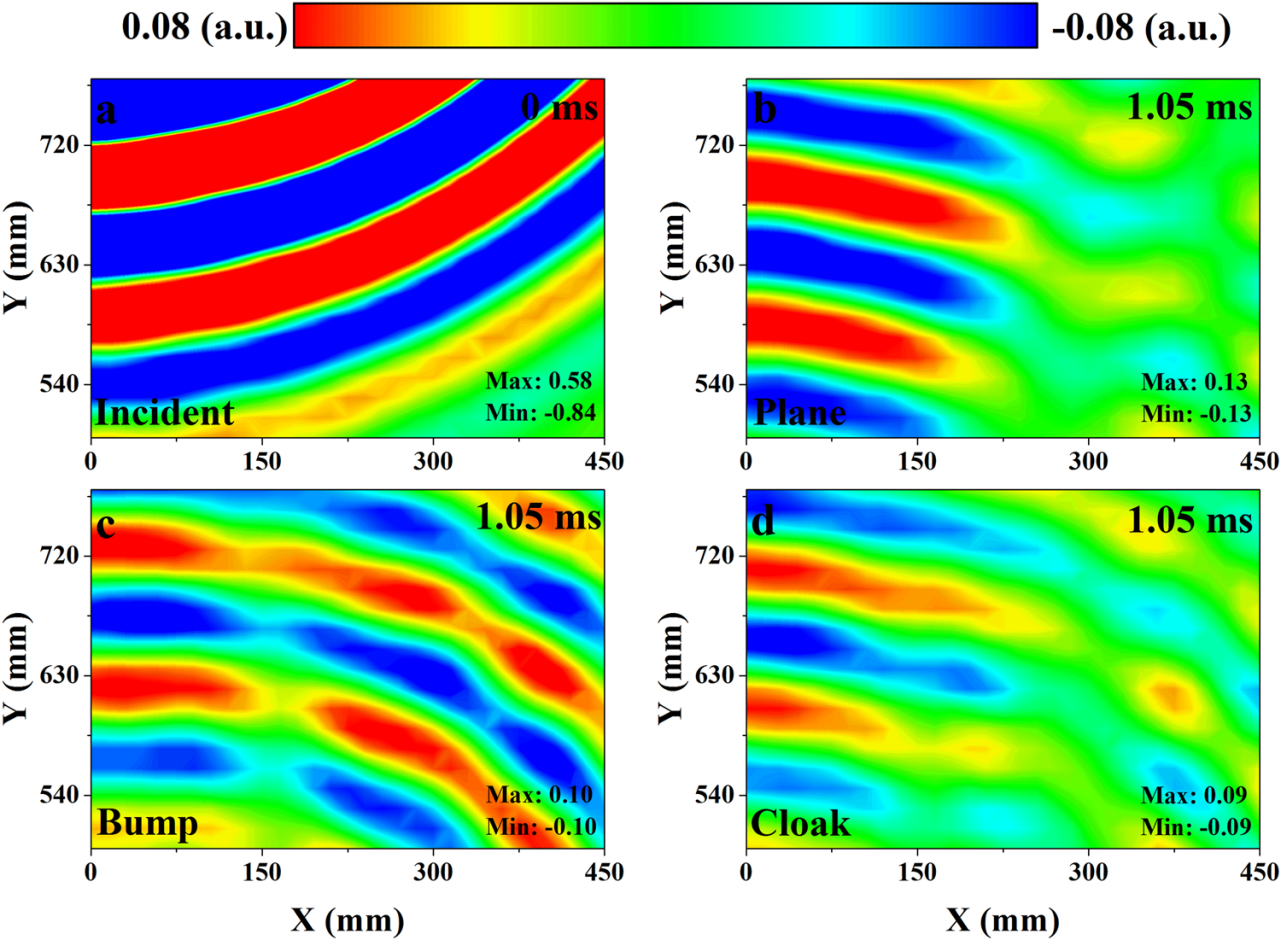
Measured acoustic pressure fields at 14 kHz. (**a**) The incident pulse produced by the source. The time of the incident acoustic field is set as 0 ms. (**b**–**d**) The scattered pressure fields measured after 1.05 ms. The short Gaussian pulse is reflected from the (**b**) soft plane, (**c**) soft bump and (**d**) soft bump covered by carpet cloak respectively.

Furthermore, similar acoustic fields are also provided when the center frequency of the Gaussian pulse is 14 kHz (Fig. 5). In Fig. 5c, after the sound wave impinges on the soft bump, the scattered wave in the left part of the acoustic field (0 < X < 150) propagates from the bottom to the top directly, while the wave in the right part of the acoustic field (150 < X < 450) propagates obliquely to the upper right. The directivity of the scattered wave implies that the acoustic signature of the soft bump is much clearer with the increase of the frequency. Figure 4b,d also show the acoustic pressure distributions obtained with the soft plane and cloaked bump, respectively. Similarly, comparing the phase, the amplitude and the propagation direction of the scattered wave in these three pressure distributions, the cloaked bump mimics the soft plane well at 14 kHz.

**Figure 6:**
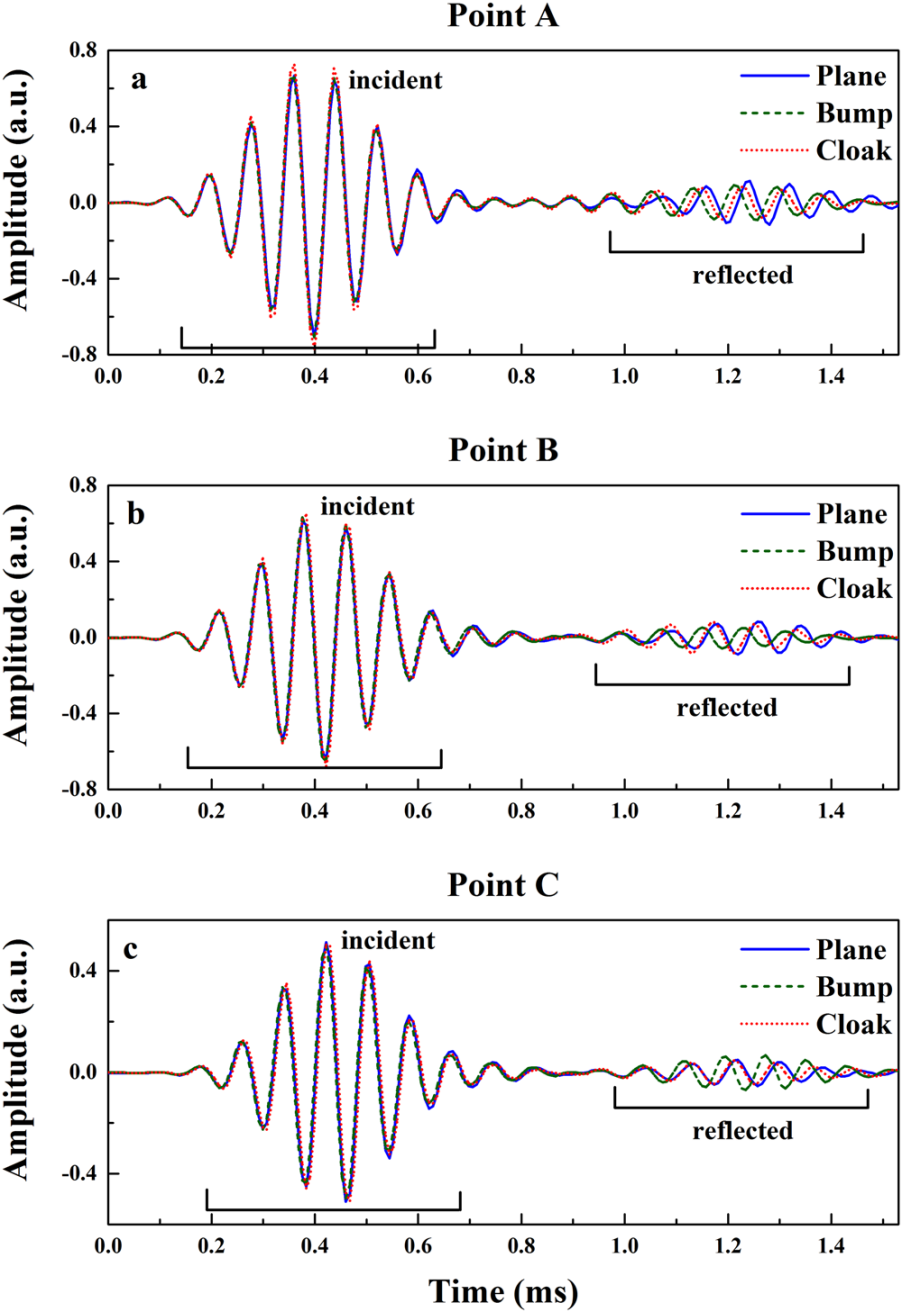
Extracted time domain signals at 12 kHz. The locations of A, B and C are specified in Fig. 3a. Each plot shows the incident wave and the scattered wave for three scenarios: the soft plane underwater (shown as blue solid lines), the soft bump underwater (shown as green dash lines), and the cloaked soft bump underwater (shown as red dot curves).

To further exhibit the performance of the cloak, we also extract the time domain signals at points A, B and C in Fig. 3a at 12 kHz (the sound pressure fields at 12 kHz can be seen in Supplementary Fig. S4). The center in the bottom of the trapezoid is set as the original point (marked as point *O* in Fig. 3a), and the coordinate of these points are A(0, 620), B(180, 620) and C(375, 620), respectively. These points are chosen for their special positions: point A is in the backscattering direction; point C corresponds to the direction where the amplitude of the wave reflected by the bare soft bump is maximum; point B is in the transitional position from point A to point C. The extracted time domain signals are shown in Fig. 6. These curves in each panel illustrate the signals obtained with the soft plane (blue solid lines), bare soft bump (green dash lines) and cloaked soft bump (red dot curves), respectively. The incident Gaussian pulses (located at about 0.4 ms) in three cases are identical, so the reflected signals (located at about 1.2 ms) can be compared with each other. The measured results at position A (Fig. 6a) show that the phase difference between the reflected waves from the cloaked bump and the soft plane is within 50° (30° at point B and 23° at point C) while the phase difference between the reflected waves obtained from the bare bump and the soft plane is about 115° (130° at point B and 110° at point C). The results also show that the carpet cloak corrects the amplitude difference. The amplitude of the scattered wave obtained from the cloaked bump is much closer to that from the soft plane. The same results can also be obtained at other frequencies (see Supplementary Figs S5 and S6).

Additionally, the reduced total radar cross section (RCS) is introduced to evaluate the camouflage effect of the cloak^43^. It is defined as:

9 $$\{\begin{array}{c}{\sigma }_{{\rm{reduced}}}=\frac{{\sigma }_{{\rm{cloaked}}}}{{\sigma }_{{\rm{uncloaked}}}}={\oint }_{{\rm{\Omega }}}(\frac{{|{P}_{cloaked,scat}|}^{2}}{{|{P}_{uncloaked,scat}|}^{2}})d{\rm{\Omega }}\\ \begin{array}{c}{P}_{cloaked,scat}={P}_{cloaked,tot}-{P}_{plane,tot}\\ {P}_{{\rm{un}}cloaked,scat}={P}_{uncloaked,tot}-{P}_{plane,tot}\end{array}\end{array},$$

where *P*_*cloaked,tot*_, *P*_*uncloaked,tot*_ and *P*_*plane,tot*_ are the total scattered field for the cloaked soft bump, the bare soft bump and the soft plane, respectively. In Eq. (9), the smaller the value is, the better the performance of the carpet cloak is.

**Figure 7:**
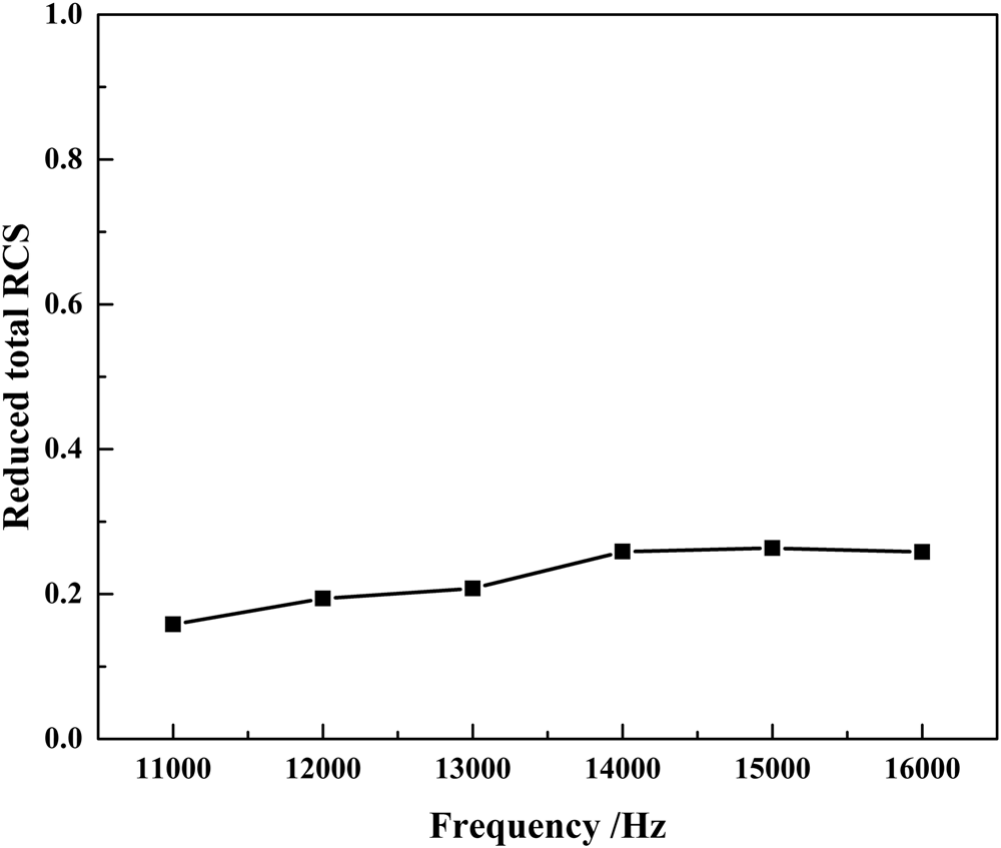
Measured reduced total RCS.

The reduced total RCSs for the measured frequencies are shown in Fig. 7. It can be observed that all the values are around 0.2. The small values demonstrate that the carpet cloak works well in a wide frequency range.

## Discussion

We realized a simple underwater carpet cloak to mimic a reflecting plane. The structure of carpet cloak, which is comprised of layered brass plates, is greatly simplified at the cost of some impedance match. The performance of the carpet cloak is assessed experimentally by measuring the acoustic pressure fields in an anechoic tank. The measured results confirm that the carpet cloak can hide the information of the bump on the reflecting plane in a wide frequency range. The proposed carpet cloak, whose unit cell size is close to one fortieth of the wavelength, shows the ability to control the underwater acoustic wave in the deep subwavelength scale. This may bring great potential engineering applications in the practical underwater devices.

## Electronic supplementary material

Supplementary Information of “Design and demonstration of an underwater acoustic carpet cloak”
