# Supplementary material for: Design and demonstration of an underwater acoustic carpet cloak
Source: Sci Rep. 2017 Apr 6;7:705. doi: 10.1038/s41598-017-00779-4 (PMC5429626; doi:10.1038/s41598-017-00779-4)
Supplement: Supplementary file 1 — Supplementary Information of “Design and demonstration of an underwater acoustic carpet cloak” [file 41598_2017_779_MOESM1_ESM.doc]

# Supplementary Information of

# “Design and demonstration of an underwater acoustic carpet cloak”

Yafeng Bi^1^, Han Jia^1,2^, Wenjia Lu^1^, Peifeng Ji^1^ and Jun Yang^1,2^

^1^Key Laboratory of Noise and Vibration Research, Institute of Acoustics, Chinese Academy of Sciences, Beijing 100190, People’s Republic of China

^2^State Key Laboratory of Acoustics, Institute of Acoustics, Chinese Academy of Sciences, Beijing 100190, People’s Republic of China

**S1. Simulated results of the real structure**

This section provides the simulated results for the designed sample at 13 kHz. In addition to the normal incidence (Fig. S1), the acoustic pressure fields for oblique incidence (30^o^ and 45^o^) are also presented in Figs. S2 and S3. To better exhibit the camouflage effect at oblique incidence, an infinite soft plane (bottom boundary) is applied instead of the soft plate.


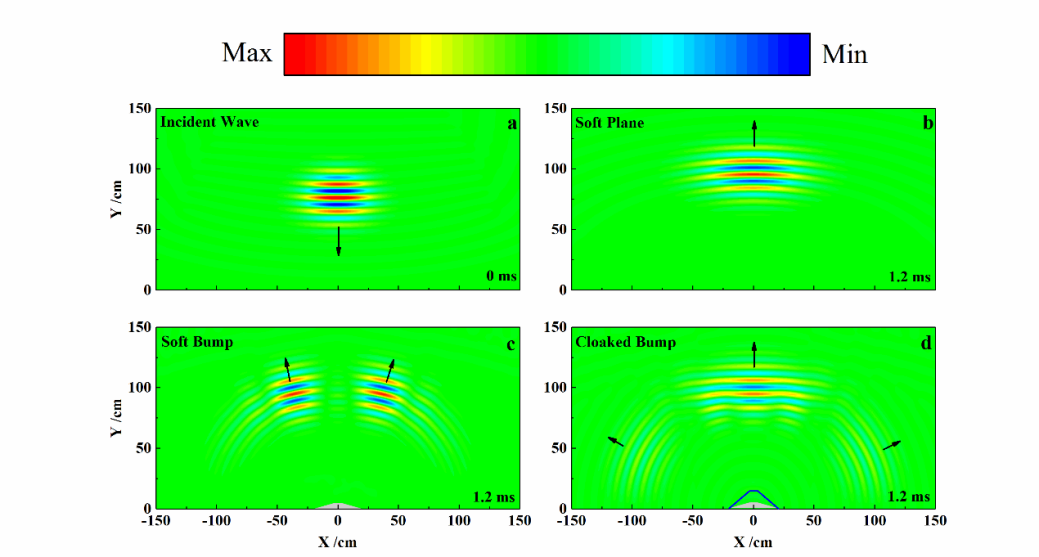


**Figure S1 | Numerical simulations of the underwater carpet cloak when the incident angle is 0^o^.** (a) The incident acoustic field. (b-d) The scattered acoustic fields. The acoustic wave is reflected by (b) a soft plane, (c) a soft bump placed on the plane and (d) a cloaked soft bump placed on the plane, respectively. Black arrows represent the propagation of the wave.


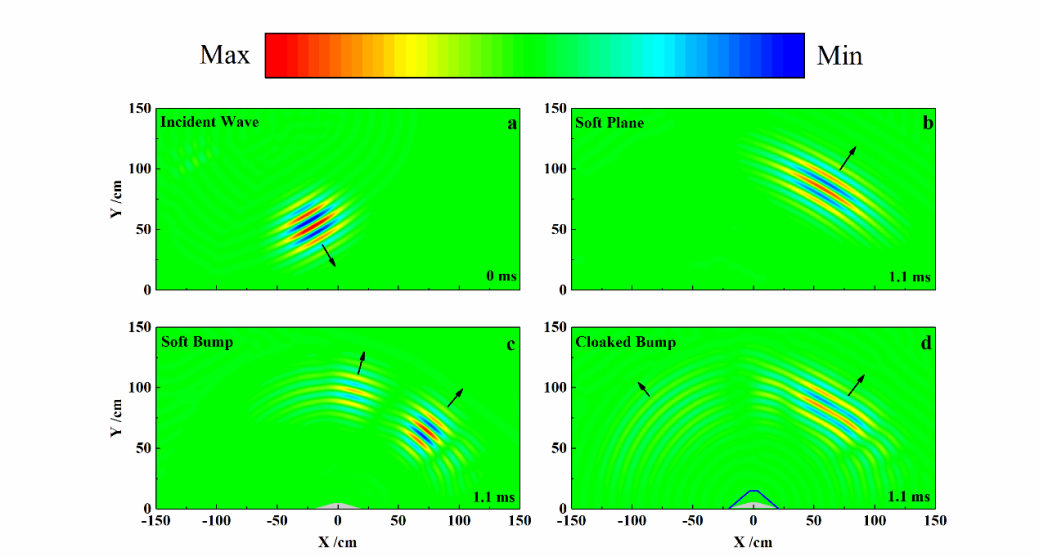


**Figure S2 | Numerical simulations of the underwater carpet cloak when the incident angle is 30^o^.** (a) The incident acoustic field. (b-d) The scattered acoustic fields. The acoustic wave is reflected by (b) a soft plane, (c) a soft bump placed on the plane and (d) a cloaked soft bump placed on the plane, respectively. Black arrows represent the propagation of the wave.


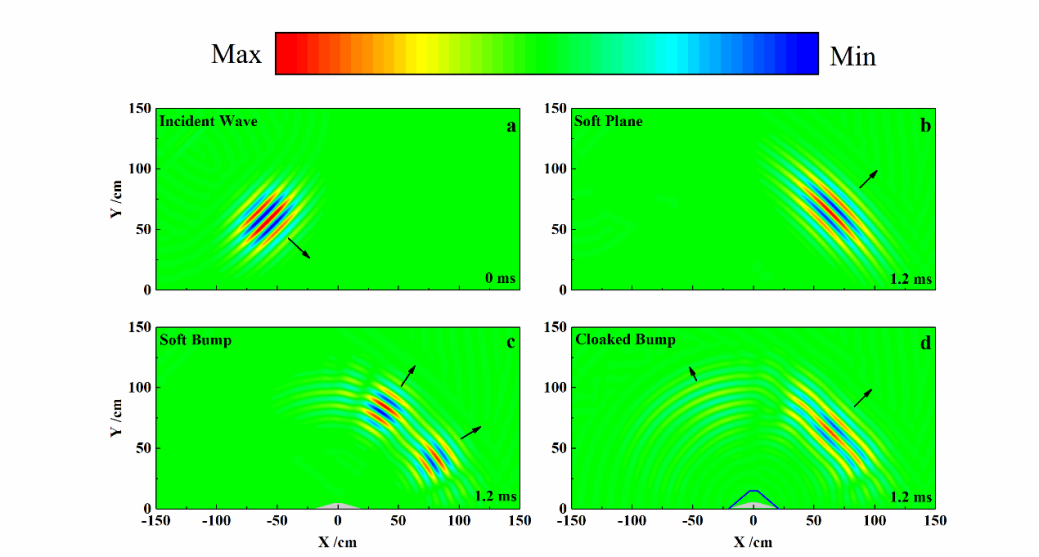


**Figure S3 | Numerical simulations of the underwater carpet cloak when the incident angle is 45^o^.** (a) The incident acoustic field. (b-d) The scattered acoustic fields. The acoustic wave is reflected by (b) a soft plane, (c) a soft bump placed on the plane and (d) a cloaked soft bump placed on the plane, respectively. Black arrows represent the propagation of the wave.

**S2.** **Measured acoustic pressure fields at 12 kHz**


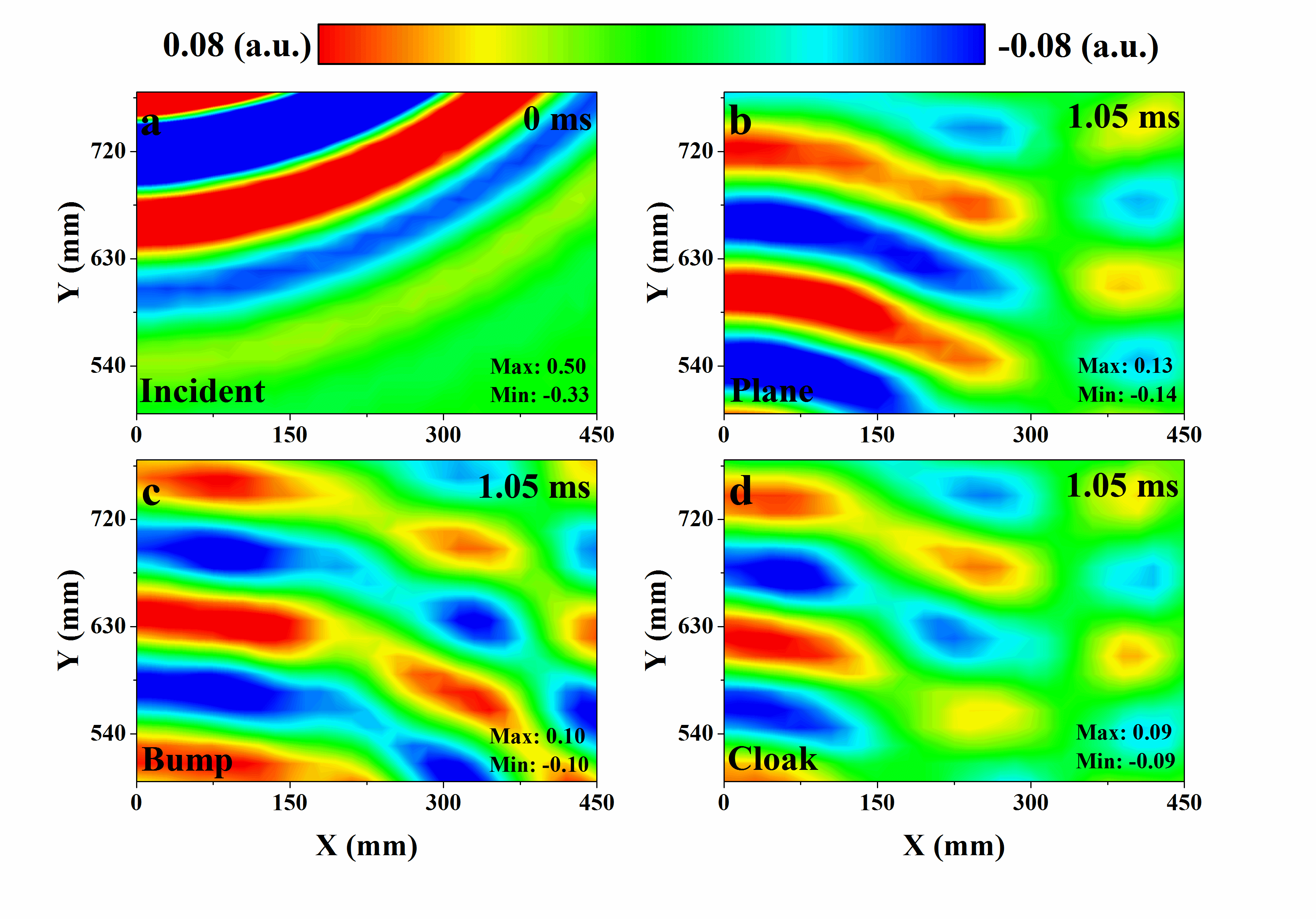


**Figure S4 | Measured acoustic pressure fields at 12 kHz.** (a) The acoustic field of the incident pulse. The time of the incident acoustic field is set as 0 ms. (b-d) The scattered pressure fields measured after 1.05 ms. The short Gaussian pulse is reflected from the (b) soft plane, (c) soft bump and (d) soft bump covered by carpet cloak respectively.

**S3. Extracted time domain signals**


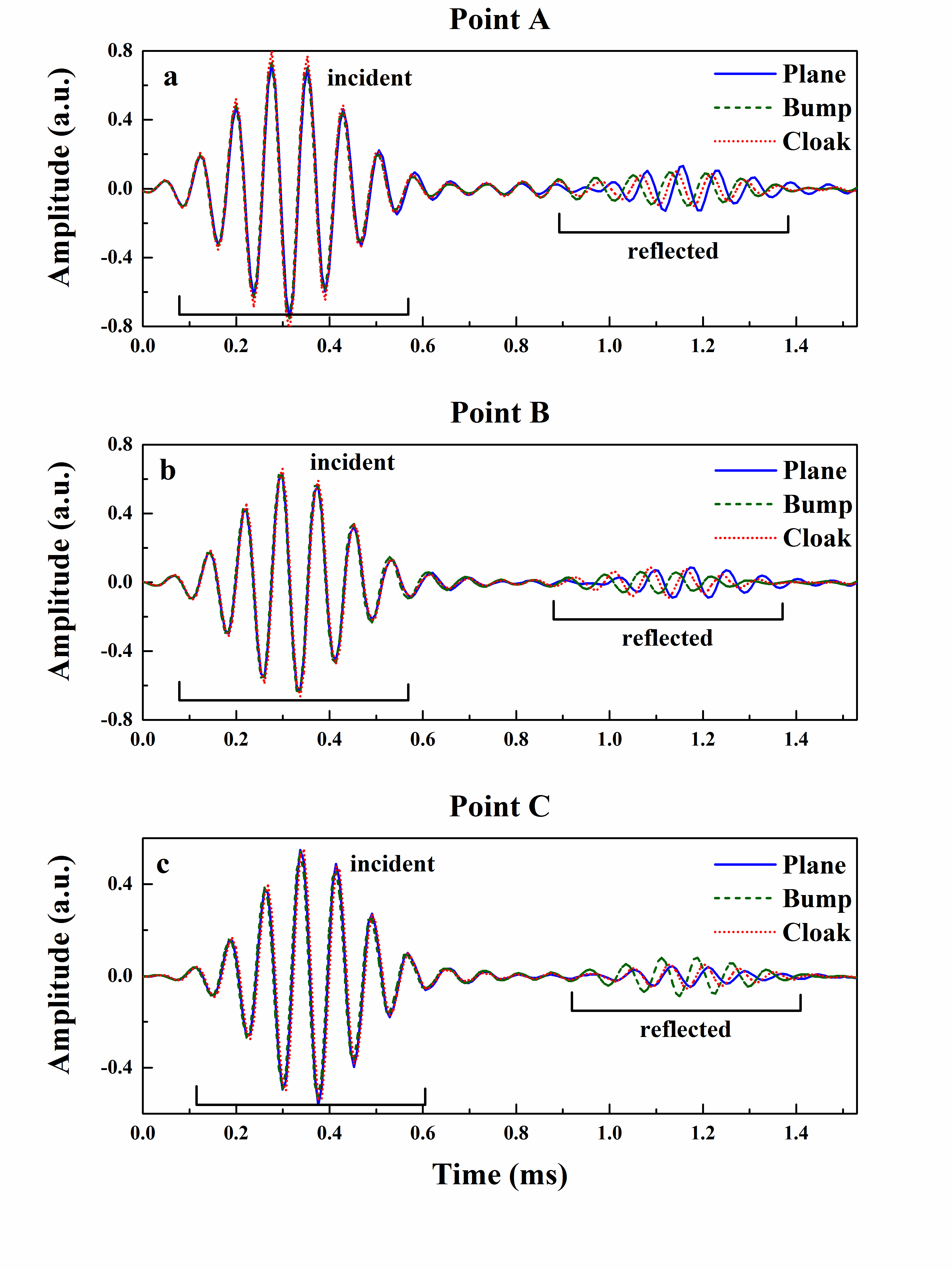


**Figure S5 | Extracted time domain signals at 13 kHz.** Each plot shows the incident wave and the scattered wave for three scenarios: the soft plane underwater (shown as blue solid lines), the soft bump underwater (shown as green dash lines), and the cloaked soft bump underwater (shown as red dot curves).


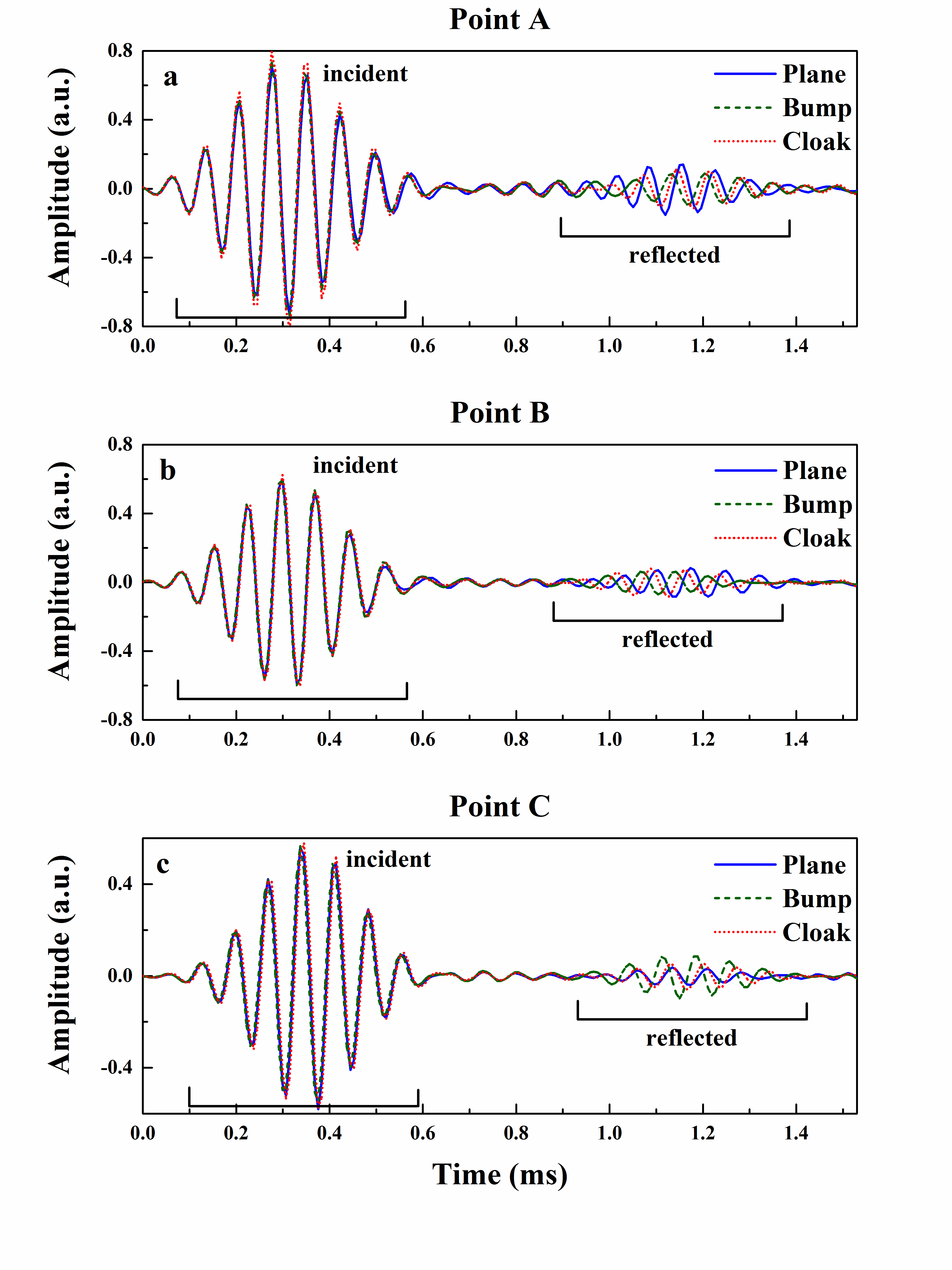


**Figure S6 | Extracted time domain signals at 14 kHz.** Each plot shows the incident wave and the scattered wave for three scenarios: the soft plane underwater (shown as blue solid lines), the soft bump underwater (shown as green dash lines), and the cloaked soft bump underwater (shown as red dot curves).
